# T1 mapping of the myocardium: intra-individual assessment of post-contrast T1 time evolution and extracellular volume fraction at 3T for Gd-DTPA and Gd-BOPTA

**DOI:** 10.1186/1532-429X-14-26

**Published:** 2012-04-28

**Authors:** Nadine Kawel, Marcelo Nacif, Anna Zavodni, Jacquin Jones, Songtao Liu, Christopher T Sibley, David A Bluemke

**Affiliations:** 1 Radiology and Imaging Sciences, National Institutes of Health, 10 Center Drive, Bethesda, MD, 20892-1074, USA; 2 Molecular Biomedical Imaging Laboratory, National Institute of Biomedical Imaging and Bioengineering, National Institutes of Health, 10 Center Drive, Bethesda, MD, 20892-1074, USA

**Keywords:** T1 mapping, Modified Look-Locker Inversion Recovery, Extracellular volume fraction, ECV, Gadobenate dimeglumine, Gadopentetate dimeglumine

## Abstract

**Purpose:**

Myocardial T1 relaxation time (T1 time) and extracellular volume fraction (ECV) are altered in patients with diffuse myocardial fibrosis. The purpose of this study was to perform an intra-individual assessment of normal T1 time and ECV for two different contrast agents.

**Methods:**

A modified Look-Locker Inversion Recovery (MOLLI) sequence was acquired at 3 T in 24 healthy subjects (8 men; 28 ± 6 years) at mid-ventricular short axis pre-contrast and every 5 min between 5-45 min after injection of a bolus of 0.15 mmol/kg gadopentetate dimeglumine (Gd-DTPA; Magnevist®) (exam 1) and 0.1 mmol/kg gadobenate dimeglumine (Gd-BOPTA; Multihance®) (exam 2) during two separate scanning sessions. T1 times were measured in myocardium and blood on generated T1 maps. ECVs were calculated as $\left(\Delta {\text{R}1}_{\text{myocardium}}/\Delta {\text{R}1}_{\text{blood}}\right)*\left(1-\text{hematocrit}\right)$.

**Results:**

Mean *pre-contrast* T1 relaxation times for myocardium and blood were similar for both the first and second CMR exam (p > 0.5). Overall mean *post-contrast* myocardial T1 time was 15 ± 2 ms (2.5 ± 0.7%) shorter for Gd-DTPA at 0.15 mmol/kg compared to Gd-BOPTA at 0.1 mmol/kg (p < 0.01) while there was no significant difference for T1 time of blood pool (p > 0.05). Between 5 and 45 minutes after contrast injection, mean ECV values increased linearly with time for both contrast agents from 0.27 ± 0.03 to 0.30 ± 0.03 (p < 0.0001). Mean ECV values were slightly higher (by 0.01, p < 0.05) for Gd-DTPA compared to Gd-BOPTA. Inter-individual variation of ECV was higher (CV 8.7% [exam 1, Gd-DTPA] and 9.4% [exam 2, Gd-BOPTA], respectively) compared to variation of *pre-contrast* myocardial T1 relaxation time (CV 4.5% [exam 1] and 3.0% [exam 2], respectively). ECV with Gd-DTPA was highly correlated to ECV by Gd-BOPTA (r = 0.803; p < 0.0001).

**Conclusion:**

In comparison to *pre-contrast* myocardial T1 relaxation time, variation in ECV values of normal subjects is larger. However, absolute differences in ECV between Gd-DTPA and Gd-BOPTA were small and rank correlation was high. There is a small and linear increase in ECV over time, therefore ideally images should be acquired at the same delay after contrast injection.

## Background

Delayed gadolinium enhancement is an established technique in cardiovascular magnetic resonance (CMR) to demonstrate *focal* myocardial scar/ fibrosis [1]. Since a correlation between *post-contrast* myocardial T1 relaxation time (T1 time) and histologically proven fibrosis has been demonstrated [2], the potential of quantitative analysis of *diffuse* myocardial fibrosis by means of CMR has been postulated [3]. Alteration of myocardial T1 times compared with values of normal subjects has been reported in association with adult congenital heart disease [4], cardiac amyloidosis [5], myocardial involvement in systemic lupus erythematosus [6], and chronic aortic regurgitation [7].

After gadolinium injection, contrast dose and type influence T1 times [8]. So far there is also no standardized method of reporting T1 times. While some authors use absolute values [3,5] others prefer the partition coefficient [9], extracellular volume fraction (ECV) [10] or fibrosis index [4]. As opposed to absolute T1 times, the ECV of myocardium is postulated to be constant over time under equilibrium conditions [11]. ECV values are calculated from the change in relaxation rate (R1 = 1/T1) of blood and myocardium corrected for the hematocrit. ECV is reported as a fraction and ranges from 0 (all fluid) to 1 (solid with no extracellular component) [11,12].

Gadopentetate dimeglumine (Gd-DTPA) is a gadolinium based contrast agent commonly used at a dose of 0.15 – 0.2 mmol/kg in CMR. [3,7] Due to a higher relaxivity [13] related to protein binding, some publications report gadobenate dimeglumine (Gd-BOPTA) at a lower dose of 0.1 mmol/kg for CMR [14,15].

The purpose of this study was to determine normal ECV and T1 values for healthy volunteers, to assess stability of ECV over time and to evaluate the influence of two contrast agents with different relaxivity on calculated ECV.

## Methods

### Study population and image acquisition

24 healthy subjects (8 men; mean age ± SD, 28 ± 6; age range 19–40) were imaged in two separate sessions using a 3T scanner (Verio, Siemens Medical Solutions, Erlangen, Germany) and a 32-channel cardiac coil. Volunteers were recruited via the Volunteer Recruitment Office of the National Institutes of Health. All study participants signed informed consent as part of an ongoing institutional review board approved study. All participants underwent two different scanning sessions for Gd-DTPA and Gd-BOPTA evaluation. Images were acquired pre-contrast and every 5 minutes between 5 and 45 minutes after injection of a bolus of 0.15 mmol/kg Gd-DTPA (Magnevist, Bayer Healthcare; concentration 0.5 mol/l) (exam 1) and 0.1 mmol/kg Gd-BOPTA (Multihance, Bracco Diagnostics; concentration 0.5 mol/l) (exam 2), respectively. The MOLLI sequence was acquired repetitively as a single mid-ventricular short axis image at the same slice position in mid- to end-diastole. Delay time varied depending on heart rate.

The aromatic ring of Gd-BOPTA enables weak plasma protein binding resulting in a higher relaxivity in plasma/blood compared to Gd-DTPA [16-18]. Several studies have demonstrated that a lower dose of Gd-BOPTA has similar diagnostic efficacy compared to Gd-DTPA at a higher dose [15,19,20]. In order to achieve somewhat similar post-contrast T1 times and to be consistent with the institutions’ practice, we used a lower dose of Gd-BOPTA (0.1 mmol/kg) compared to Gd-DTPA (0.15 mmol/kg). These dose values have also previously been used in the literature [3,11,14,15]. Both contrast agents were injected intravenously at 2 ml/s using a power injector and followed by a 30 ml saline bolus administered at the same flow rate. The short MOLLI sequence used in the current study was adapted from that introduced by Messroghli et al. and has been described previously [21,22]. Short MOLLI acquired 8 images at different inversion times using the following scan parameters: TE/TR 1.03/2.4 ms; flip angle 35°; bandwidth 1002 Hz/Px; minimum TI 125 ms; TI increment 80 ms; image acquisition time 217 ms; FOV 360 × 288 mm^2^; pixel size 2.3 × 1.9 mm^2^ (interpolated to 1.2 × 1.0 mm^2^); slice thickness 8 mm; iPAT factor (GRAPPA) 2.

All study participants underwent 12 lead ECG as well as a history and physical examination prior to each CMR study. All study participants were determined to be free of clinical cardiovascular or systemic disease and had a normal electrocardiogram. A CMR examination performed prior to the current study showed normal global and regional function. Myocardial late gadolinium enhancement imaging did not show a myocardial scar in any of the subjects. Table 1 shows further characteristics of the study subjects.

**Table 1:** Volunteer (n = 24) characteristics for both exams

**Characteristics**	**Gd-DTPA**	**Gd-BOPTA**
	(exam 1)	(exam 2)
Age in years, mean ± SD	28 ± 6	
Male, n (%)	8 (33)	
Weight in kg, mean ± SD	69.0 ± 12.7	69.0 ± 13.1
Hematocrit in %, mean ± SD	39.8 ± 3.9	39.5 ± 3.9
Heart rate in bpm*, mean ± SD	62 ± 8	61 ± 12
Creatinine in mg/dl, mean ± SD	0.8 ± 0.2	0.8 ± 0.2
Gadolinium dose in ml, mean ± SD	20.4 ± 4.1	13.9 ± 2.6

*during the MOLLI sequence prior to contrast injection.

### Image analysis

**Figure 1:**
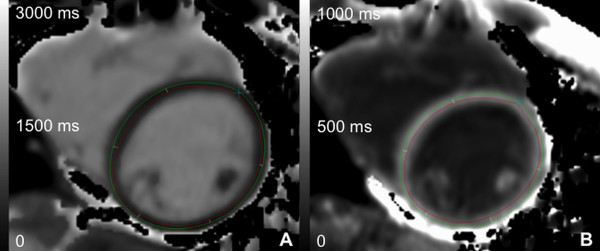
**T1 maps with measurements.** T1 map pre- (A) and post-contrast (B) with left ventricular endocardial and epicardial contours.

T1 maps were generated using MRmap [23]. Manual motion correction was performed when necessary. Pre-contrast T1 relaxation time acquired at 3T is longer and might not fully recover when the same sampling schemes are used compared to 1.5T. In a phantom study recently published it has been demonstrated that for a heart rate of 60 bpm, the difference between MOLLI- and inversion-recovery spin-echo (IR-SE) -derived T1 times were smaller than 5% for a T1 time less than 500 ms. The difference increased to 5-10% for T1 times between 500-1500 ms, and the difference continued to increase for T1 times of more than 1500 ms [22]. Further, underestimation of T1 time increases with increasing heart rate. Therefore in the current study heart rate correction was performed for pre-contrast T1 times of myocardium and blood. The heart rate correction algorithm was based on the phantom data published previously by Lee et al. [22]. MOLLI-derived T1 relaxation times of different heart rates were fitted to IR-SE-derived T1 relaxation times using 2nd order polynomial fitting. An Additional file 1 shows in detail how heart rate correction was performed [see Additional file 1. Pre-contrast in vivo MOLLI –derived T1 relaxation times were corrected using this function. T1 maps were transferred to QMass V.7.2 (Medis Medical Imaging Systems, Netherlands). Left ventricular endocardial and epicardial contours were drawn manually while segments were defined automatically by the software after marking the border between segment 7 and 8 (Figure 1). T1 times at the mid short axis slice of the left ventricle were determined for each segment (American heart association [AHA] segments 7–12) [24]. Segments with severely impaired image quality related to primarily motion artifacts involving the whole segment were identified. An overall myocardial T1 time for each slice was calculated, excluding segments with severely impaired image quality. T1 time of the blood pool was measured by manually drawing a region of interest in the blood pool of the left ventricular cavity excluding papillary muscles. For 10 randomly chosen subjects, measurements were performed by a second independent reader to assess inter-observer variability. The ECV was calculated as $\Delta {\text{R}1}_{\text{myocardium}}/\Delta {\text{R}1}_{\text{blood}}*\left(1-\text{hematocrit}\right)$, where ΔR1_myocardium_ = 1/T1_myocardium pre contrast_ - 1/T1_myocardium post contrast_ and ΔR1_blood_ = 1/T1_blood pre contrast_ - 1/T1_blood post contrast_[12].

### Statistical analysis

Statistical analysis was performed using SPSS (IBM statistical software; version 19). Continuous variables are expressed as mean ± SD. *Pre-contrast* myocardial T1 times for Gd-DTPA and Gd-BOPTA were compared using a paired t-test. *Post-contrast* T1 times were analyzed using linear mixed-model analyses with separate models incorporating the log transformed T1 times for blood and myocardium as the dependent variable, including the contrast agent and time as the predictors. Linear mixed-model analysis of the ECV values as the outcome with contrast type and time as independent variables was also performed in order to identify group related differences. After adjustment of ECV values for time, nonparametric analysis was used to assess the correlation/ rank of ECV for Gd-DTPA versus Gd-BOPTA using Spearman’s correlation coefficient. Bland-Altman plots were generated for each time point *after contrast* injection to compare ECV of exam 1 and exam 2. A p-value of <0.05 was considered to be statistically significant. To assess inter-individual variation the coefficient of variation (CV) was calculated for T1 relaxation time of myocardium and blood *before* and *after contrast* injection and for ECV values.

To compare inter-observer agreement, the intraclass correlation coefficient (ICC) using a two-way random model (ICC < 0.40 = poor; ICC ≥ 0.40 to 0.75 = fair to good; ICC > 0.75 = excellent agreement) was calculated.

## Results

672T1 maps at the pre-specified time points were available for evaluation; 4 T1 maps were not available due to scanner malfunction. Due to severely impaired image quality 52/4032 (1.3%) segments were excluded from further analysis. The mean time between intra-individual repeat scans was 51 ± 34 days.

### Normal T1 relaxation time of myocardium and blood: Gd-DTPA and Gd-BOPTA

As expected, *prior to contrast injection*, mean T1 relaxation times for myocardium were similar for both the first and second CMR exam (1286 ± 59 and 1273 ± 39, respectively, p = 0.2). Also, blood T1 times were similar at exams 1 and 2 (2074 ± 139 and 2097 ± 112, respectively, p = 0.4).

**Figure 2:**
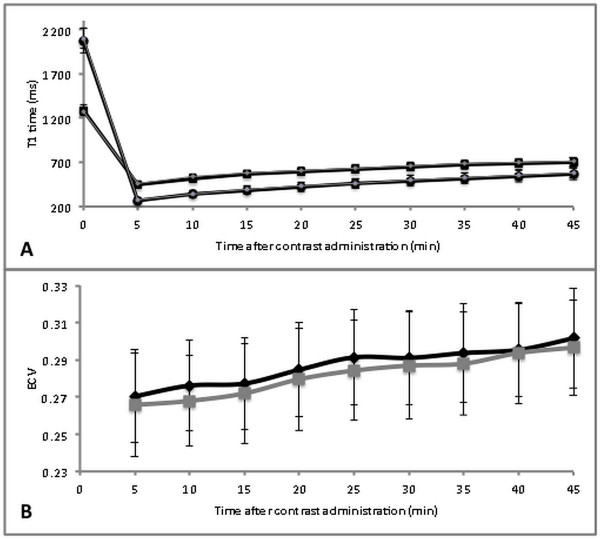
**Change in mean T1 time ± SD over time.** Change in T1 time of myocardium and blood (A) and ECV (B) over time. Black square (A) = myocardium Gd-DTPA; black circle (A) = blood Gd-DTPA; grey diamond (A) = myocardium Gd-BOPTA; grey circle (A) = blood Gd-BOPTA; black diamond (B) = ECV Gd-DTPA; grey square (B) = ECV Gd-BOPTA.

Figure 2A shows the mean ± SD of T1 relaxation times for myocardium and blood *pre-contrast* and over time between 5 and 45 minutes *after contrast injection* for exams acquired with Gd-DTPA (exam 1) and Gd-BOPTA (exam 2). The mean myocardial T1 relaxation time was 15 ± 2 ms (2.5 ± 0.7%) lower for Gd-DTPA compared to Gd-BOPTA (p < 0.01) averaged over all time points *after contrast* injection (5 min to 45 min). For blood pool, T1 relaxation time was not significantly different between Gd-DTPA and Gd-BOPTA (p > 0.05).

Coefficient of variation (CV) of *pre-contrast* T1 relaxation time of myocardium for exam 1 was 4.5% and for exam 2 3.0%. Overall CV of *post-contrast* myocardial T1 relaxation times averaged over all time points was 7.0% (exam 1, Gd-DTPA) and 5.9% (exam 2, Gd-BOPTA). CV of T1 relaxation times measured in blood *pre-contrast* was 6.5% (exam 1) and 5.5% (exam 2), respectively. CV of *post-contrast* T1 relaxation time of blood averaged over all time points was 12.2% (exam 1, Gd-DTPA) and 9.5% (exam 2, Gd-BOPTA), respectively.

### Normal ECV values: Gd-DTPA and Gd-BOPTA

**Figure 3:**
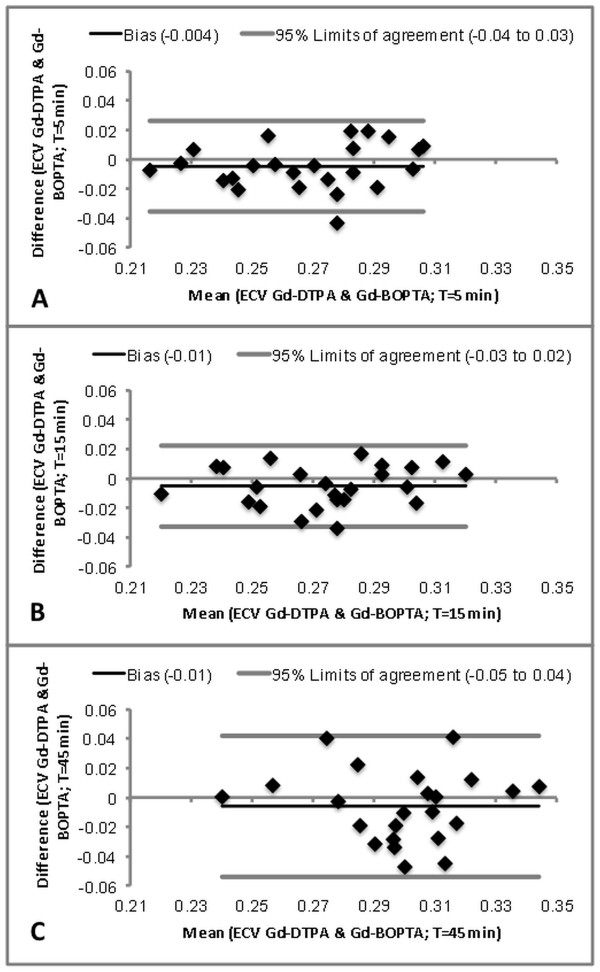
**Bland-Altman plots comparing ECV values of exam 1 (Gd-DTPA) and exam 2 (Gd-BOPTA).** Bland-Altman plots of the acquisitions at 5 min (A), 15 min (B), and 45 min (C) are shown exemplarily.

On average, ECV of Gd-DTPA was slightly greater (by 0.01) than that of Gd-BOPTA between 5 and 45 min (p < 0.05). The observed range of the normal ECV values averaged over all time points was about 30% of the mean value. However, subjects with higher ECV values for Gd-DTPA were also the subjects who tended to show higher ECV values for Gd-BOPTA (Spearman’s correlation coefficient = 0.803, p < 0.0001). Bland-Altman plots demonstrated a good agreement of ECV values obtained in exam 1 and exam 2 for all time points (Figure 3). CV of ECV was 8.7% (exam 1, Gd-DTPA) and 9.4% (exam 2, Gd-BOPTA), respectively.

Mean ECV of both gadolinium agents (Gd-DTPA and Gd-BOPTA) increased linearly over time (p < 0.0001) (Figure 2B). Between the 5 to the 45 minute acquisition, mean values for both contrast agents increased from 0.27 ± 0.03 to 0.30 ± 0.03. In practice, ECV values will likely be obtained at only 1 time point after gadolinium injection. Thus, the regression equations between time and ECV values yielded the following time correction factors:

(1) $$\begin{array}{c} {\text{ECV}}_{\text{calc}}{\text{=ECV}}_{\text{acq}}-0.0007\left({\text{t}}_{\text{acq}}{-\text{t}}_{\text{calc}}\right)\;\left(\text{for Gd-DTPA at a dose of}\;0.15\;\text{mmol/kg}\right)\\ {\text{ECV}}_{\text{calc}}{\text{=ECV}}_{\text{acq}}-0.0008\left({\text{t}}_{\text{acq}}{-\text{t}}_{\text{calc}}\right)\;\left(\text{for Gd-BOPTA at a dose of}\;0.1\;\text{mmol/kg}\right) \end{array}$$

where t_acq_=time (in minutes) after contrast injection when MOLLI was acquired, t_calc_=time (in minutes) after contrast injection of interest, ECV_acq_=ECV at the time point t_acq_ and ECV_calc_=ECV at the time point t_calc_.

### Inter-observer agreement

Inter-observer agreement was excellent for measurements of T1 relaxation time for myocardium and blood. ICC for myocardium was 1.0 (exam 1, Gd-DTPA) and 0.997 (exam 2, Gd-BOPTA), respectively. ICC for blood was 0.999 (exam 1, Gd-DTPA) and 0.998 (exam 2, Gd-BOPTA).

## Discussion

The current study evaluates the range of normal values for T1 time and ECV at 3 Tesla for two in CMR commonly used gadolinium containing contrast agents, namely Gd-DTPA at a dose of 0.15 mmol/kg and Gd-BOPTA at 0.1 mmol/kg for an ethnically diverse group of young adults (≤ 40 years). Differences in ECV between the two contrast agents were small but significant (mean difference, 0.01). However, the range of normal ECV’s varied by approximately one third of the mean value. In addition, healthy subjects who had higher ECV values for Gd-DTPA also ranked higher in their ECV values for the Gd-BOPTA examination, performed on average 51 ± 34 days later. ECV showed a small but nearly linear increase over time between 5 and 45 minutes after both Gd-DTPA and Gd-BOPTA.

### T1 times and ECV of Gd-DTPA versus Gd-BOPTA

It is known that T1 relaxation time of a given tissue is longer at 3T compared to 1.5T [25]. This explains why myocardial *pre-contrast* T1 times acquired in the current study are longer compared to previous publications at 1.5T [3]. It has been demonstrated that particularly at high heart rates and long T1 relaxation times MOLLI underestimates true T1 time [22]. Therefore in the current study *pre-contrast* T1 times were corrected for heart rate. This explains why average myocardial *pre-contrast* T1 relaxation time is about 10% longer compared to values reported by Piechnik et al.[26]. Compared to Lee et al. values are 2% shorter, which might be explained by the higher mean age of 36 ± 13 years in the afore mentioned study compared to 28 ± 6 years in the current study [22]. Diffuse fibrosis is known to occur with “normal aging” of the heart and a correlation between age and the extracellular volume fraction has already been demonstrated [27].

Myocardial T1 relaxation times have been demonstrated to correlate with histologically proven fibrosis [2] and correlation between ECV and histological fibrosis was shown [10]. Although T1 relaxation times of equimolar doses of Gd-BOPTA are significantly lower compared to Gd-DTPA, differences in ECV have not previously been determined. After dose adjustment, in the current study we observed small but slightly higher ECV values for Gd-BOPTA compared to Gd-DTPA by .01 (mean difference, or approximately 4% of the mean values) (Figure 2B). The binding of Gd-BOPTA to human serum albumin results in a lower molecular tumbling rate of the molecule and a longer rotational MR correlation time leading to an increased relaxivity [28]. Since albumin is mainly present in blood, the distribution between blood and myocardium is expected to be different between Gd-BOPTA and Gd-DTPA whereas pre-contrast values are equal. This should affect the ratio of the change in relaxation rate of myocardium and blood and might explain the difference in ECV values.

### Variation in ECV over time

Calculation of ECV values is based on the assumption of a two-compartment model. Due to a rapid exchange, a steady state with equal contrast concentration in the intravascular compartment and the interstitial compartment is supposed to be established [11,16,29]. There is ongoing debate whether equilibrium can only be achieved by a continuous contrast infusion technique or if a single bolus is sufficient to establish equilibrium within a certain time after contrast injection [27,30,31].

Our results show the two compartment model may be limited in that there was a continuous increase of ECV values over time for both Gd-DTPA and Gd-BOPTA after bolus injection of contrast (Figure 2B). Schelbert et al. also reported a small but statistically significant change of Ve (extravascular extracellular volume fraction) after bolus infusion of gadolinium. In their study, the increase over 30 minutes was 0.6%; in the current study, the increase over 45 minutes was about 0.03 (11%) for both agents. There are several possible explanations for the variation in magnitude of ECV increase over time between the two studies: First, the group of volunteers in the current study consisted only of healthy young adults. Schelbert et al. included a heterogeneous group of 10 study subjects only, including four older volunteers (66–81 years) with “significant comorbidity” and potentially reduced renal function (lower limits of normal) and subsequently slower contrast excretion in comparison to young healthy subjects. Second, Schelbert et al realized a significant increase in Ve over 30 minutes whereas in the current study the increase of 11% was realized between minute 5 and 45 after contrast injection. Since ECV increases linearly over time, the increase is larger with increasing duration of the observation period. And finally in the afore mentioned study it is not reported whether the increase of 0.6% was absolute or relative. Since Ve and ECV, respectively is a ratio/percentage results can be reported in different ways, what might lead to confusion. In the current study the absolute increase in ECV till minute 45 was 0.03 or 3%, relative increase was 11%.Ugander et al describe that in their study ECV remained stable over time after contrast injection. Apparently comparison was performed only between values of one time point and the following but not over the entire observation period of 25 min only [32]. In the figure that is supposed to support this statement, range of the y-axis is large (0-100%) and a smaller increase in ECV over time such as in the current study might not become obvious. Further, Ugander et al included only 11 volunteers in this part of their analysis whose age range is not mentioned.

A possible reason for the lack of equilibrium is that renal clearance might be faster than exchange rate between intravascular and interstitial compartment. Further, gadolinium based contrast agents also penetrate into other spaces such as synovial fluid and bone hampering maintenance of equilibrium [33-35]. Clinically, an increase in ECV over 45 minutes may be relevant if the MOLLI sequence is not acquired at identical post-contrast delay times. Broberg et al. measured a “fibrosis index” of 0.32 ± 0.05 in patients with different types of congenital heart disease and an index of 0.25 ± 0.02 in normal controls [4]. Messroghli et al. examined myocardial T1 times in rats before and after angiotensin II infusion and measured mean ECV values of 0.17 and 0.23, respectively [10]. In both studies mean differences were in the order between 0.06 and 0.07. Since inter-individual variation was rather high it seems important to measure ECV values at consistent times after bolus injection.

### Inter-individual variation

We demonstrated that concordance (or rank order) of ECV values between exam 1 (Gd-DTPA) and exam 2 (Gd-BOPTA) was high. For example, those subjects with a relatively higher ECV value for Gd-DTPA also had a relatively higher ECV value for Gd-BOPTA. This concordance between ECV values over the two exams was reflected by a high Spearman’s correlation coefficient of 0.803 (p < 0.0001, assessed over all time points). Blant-Altman plots (Figure 3) further demonstrate a good agreement between exams but a large inter-individual variation of ECV.

Inter-individual variation of ECV was higher (CV 8.7% [exam 1, Gd-DTPA] and 9.4% [exam 2, Gd-BOPTA], respectively) compared to variation of *pre-contrast* myocardial T1 relaxation time (CV 4.5% [exam 1, Gd-DTPA] and 3.0% [exam 2, Gd-BOPTA], respectively). According to previous publications the mean difference between maximum and minimum ECV values averaged over all time points in healthy subjects was approximately 0.09 for Gd-DTPA and 0.11 for Gd-BOPTA [27]. The somewhat broad range of ECV must be considered when identifying a cut-off or threshold value for abnormally increased or decreased ECV.

Since there was a high correlation and good agreement of ECV values between exam 1 and 2 we conclude that the large inter-individual variation in ECV values is a real finding and not related to noise. Further, Schelbert et al. also reported a large inter-individual variation of the extravascular extracellular volume fraction (Ve) [27]. The large inter-individual variation of ECV is most probably a consequence of the large inter-individual variation of the *post-contrast* T1 relaxation time of blood. Variation in post-contrast T1 time of blood is most likely related to inter-individual differences in hydration, renal excretion and distribution of contrast into other spaces such as synovial fluid and bone.

There are several limitations of this study. Our T1 times and ECV values are for younger individuals scanned at 3T. The pharmacokinetics of these gadolinium contrast agents may vary for older subjects or individuals with renal dysfunction. Given the somewhat large range of ECV values relative to the mean values in this normal population, the study size (n = 24) yields only an approximation to the 95% confidence intervals of the population. The normal values given are valid only for the doses of gadolinium that were studied (0.10 mmol/kg for Gd-BOPTA and 0.15 mmol/kg for Gd-DTPA). Ideally order of exams should be random. Given our results, intra-individual reproducibility seems to be an important parameter that should be established for planning of longitudinal studies. To test intra-individual reproducibility ideally volunteers should be scanned twice with the same protocol, contrast dose and type.

## Conclusions

Normal ECV values are affected by several factors: First, there is a large inter-individual variation of about 30% of the mean. The smaller inter-individual variation in *pre-contrast* T1 times and the ability to discriminate between normal individuals and a disease population with diffuse myocardial fibrosis requires further analysis. Second, there is a small but significant linear increase in ECV with time in the first 45 minutes after a bolus of gadolinium of 11% necessitating of either careful attention to imaging delay time or post-hoc correction for this variability to permit serial comparison. And third, there is a minor but significant difference with contrast agent of 2% between Gd-DTPA at 0.15 mmol/kg and Gd-BOPTA at 0.1 mmol/kg. However, the ECV values of Gd-BOPTA and Gd-DTPA were quite similar, and subjects with high (or low) ECV values had similar values with both contrast agents over a mean follow-up period of almost 2 months. Subsequent comparisons to patients with cardiomyopathies will be necessary to determine the clinical utility of these surrogate markers of myocardial fibrosis.

## Competing interests

The authors declare they have no competing interests.

## Authors’ contribution

NK: study design, data acquisition, data analysis, data interpretation, manuscript drafting; MN: study desing, data analysis, manuscript revision; AZ: data acquisition, data analysis, data interpretation, manuscript revision; JJ: data acquisition, manuscript revision; SL: study design, data acquisition; CS: study design, manuscript revision; DB principal investigator, study design, data interpretation, manuscript revision. All authors read and approved the final manuscript.

## Supplementary Material

Additional file 1 **Heart rate correction.** Detailed description of heart rate correction of pre-contrast T1 times. Click here for file
