# Supplementary material for: T1 mapping of the myocardium: intra-individual assessment of post-contrast T1 time evolution and extracellular volume fraction at 3T for Gd-DTPA and Gd-BOPTA
Source: J Cardiovasc Magn Reson. 2012 Apr 28;14(1):26. doi: 10.1186/1532-429X-14-26 (PMC3405486; doi:10.1186/1532-429X-14-26)
Supplement: Additional file 1 — Heart rate correction. Detailed description of heart rate correction of pre-contrast T1 times. [file 1532-429X-14-26-S1.doc]

**Heart rate correction**

The heart rate correction algorithm was based on the phantom data published previously by Lee et al . MOLLI-derived T1 relaxation times of different heart rates were fitted to inversion recovery (IR-SE) -derived T1 relaxation times using 2nd order polynomial fitting.

Below the 2nd order polynomial fitting formula for heart rates between 40bpm and 100bpm, where T1-hr is the heart rate adjusted T1 time and T1 is the native T1 time.  For heart rates that were not multiples of 10, a linear weighted interpolation was used. For example: T1-hr(45bpm) = 0.5*T1-hr(40bpm) + 0.5*T1-hr(50bpm).

HR=40, T1-hr = 0.0000209*(T1)^2^ + 1.09*(T1) - 18.75

HR=50, T1-hr = 0.0000331*(T1)^2^ + 1.086*(T1) - 22.63

HR=60, T1-hr = 0.0001064*(T1)^2^ + 0.985*(T1) - 1.93

HR=70, T1-hr = 0.0001425*(T1)^2^ + 0.9415*(T1) + 6.56

HR=80, T1-hr = 0.0002317*(T1)^2^ + 0.825*(T1) + 34

HR=90, T1-hr = 0.0003708*(T1)^2^ + 0.6336*(T1) + 79.1

HR=100, T1-hr = 0.0004255*(T1)^2^ + 0.60128*(T1) + 81.05

1. Lee JJ, Liu S, Nacif MS, Ugander M, Kawel N, Sibley CT, Kellman P, Arai A, Bluemke DA: **Myocardial T1 and Extracellular Volume Fraction Mapping at 3 Tesla.** *Journal of cardiovascular magnetic resonance : official journal of the Society for Cardiovascular Magnetic Resonance* 2011, **13:**75.
